# Magnitude of digital adaptability role: Stakeholder engagement and costless signaling in enhancing sustainable MSME performance

**DOI:** 10.1016/j.heliyon.2024.e33484

**Published:** 2024-06-22

**Authors:** Uli Wildan Nuryanto, Basrowi, Icin Quraysin, Ika Pratiwi

**Affiliations:** a Postgraduate Program, Master of Management, Bina Bangsa University, Indonesia; b Faculty of Economics and Business, Bina Bangsa University, Indonesia

**Keywords:** takeholder engagement, Costless signaling, Magnitude of digital adaptability, Sustainable MSME performance

## Abstract

This study investigates the relationship between stakeholder engagement, costless signaling, digital adaptability, and sustainable performance in micro, small, and medium enterprises (MSMEs). A quantitative research approach was employed, collecting data through questionnaires from respondents who were owners or representatives of MSMEs. Statistical analysis was conducted to test the research hypotheses. The findings revealed a significant positive impact of stakeholder engagement and costless signaling on sustainable performance in MSMEs. However, digital adaptability did not moderate the relationship between costless signaling and sustainable performance. The implications of this study suggest that MSMEs should prioritize stakeholder engagement and employ effective costless signaling strategies to enhance their sustainable performance. Support from the government and financial institutions is also crucial in facilitating these efforts. The study has limitations in terms of generalizability, and further research is needed with a broader scope and more in-depth methods to deepen the understanding of the influence of digital adaptability in the context of MSMEs.

## 1 Introduction

Entrepreneurship is seen to provide several benefits for both society and the government [1,2]. Entrepreneurial methods may create money while also increasing innovation and creativity in society [3]. On the other hand, the government encourages individuals to become entrepreneurs since this activity may boost the country's GDP and give work possibilities for educated and uneducated citizens [4]. Furthermore, starting a firm does not directly generate income, raise GDP, or provide job possibilities [5]. To achieve these objectives, a company must expand dramatically [6,7]. Regarding expansion, entrepreneurial growth is inextricably linked to the supporting components entrepreneurs receive, such as government policies ([8]; H [9]), business environment [10,11], skills [12,13], financial aid ([14]; B. [15,16]), costless signaling [[17], [18], [19]], magnitude digital of adaptability [[20], [21], [22]] and so on. Entrepreneurs can build their enterprises if they have access to this assistance. On the other hand, starting a firm does not directly generate income, raise GDP, or provide job possibilities. To achieve these objectives, a company must expand dramatically. Regarding expansion, entrepreneurial growth is inextricably linked to the supporting components entrepreneurs receive, such as government policies, business climate, skills, financial aid, and so on.

The sustainability of MSMEs, particularly those in the food and beverage industry, is dependent on the choice of their specific business growth plans [[23], [24], [25]]. The selected growth plan must be compatible with the company's internal and external environments [26]. According to Lozano et al. [27], the company's internal environment encompasses all of the company's resources as well as the company's ability to manage these resources. These resources encompass all assets, expertise, people, information, organizational procedures, capabilities, and actions that organizations may do, such as marketing, sales, and promotions [28]. Meanwhile, the company's external environment consists of economic, political, social, and technological rules in the nation where the firm works [29]. External environments are just as significant as interior environments ([30]; W. [31,32]). The external environment might influence individuals' purchasing patterns for various items or services [[33], [34], [35]]. Furthermore, food and beverage MSMEs must deal with market unpredictability and volatility [23,36]. Uncertainty and market instability generated by short product life cycles, short product design cycles, new technologies, unexpected product/service entrance, incumbent repositioning, and radical redefining [37].

Appropriate strategic decisions can be established based on this internal and external context [38]. These strategic stages will be carried out with the help of internal resources, and plans must be executed in the external environment [39]. Most organizations' popular tactics involve marketing innovation and pursuing competitive advantage, promotion, and price [39]. Vieira & Amaral [39] highlighted that integrating internal and external R&D initiatives and minimizing internal costs and time are all regarded strategic steps in the pursuit of growth. However, development is one of many factors that MSMEs must address to thrive in the business [40]. Another critical issue that MSMEs must address is the challenge that stems from both the internal and external environment [[41], [42], [43], [44]] define that internal barriers to preserving the company life cycle and surviving in the sector include a lack of technological capabilities, a lack of understanding of business development through e-commerce, and a lack of ownership of international and national certifications. Finally, external environmental constraints might arise from legislation, infrastructure, and market access [45].

Studies on the stakeholder engagement influence, especially government's role on entrepreneurial activity have risen in the previous several decades [[46], [47], [48]]. Because entrepreneurship is classified as diverse, laws and regulations are required to grow and reinforce its social position [49]. Because of its unavoidable role in the entrepreneurial process, the government is regarded as the backbone of entrepreneurial activity [50]. Every country's government knows the numerous advantages of establishing and expanding the number of MSMEs [[51], [52], [53]]. As previously said, MSMEs benefit national progress, including employment, taxation, and foreign exchange [[54], [55], [56], [57]]. As a result, rules and help are widely available for all business sizes, from micro to small and medium firms, to reap the most benefits [58,59] state that the government's effect on MSMEs is proportional to their authority in the entrepreneurial environment. Xu et al. [15] expand on this assertion, stating that the government fosters MSMEs by giving access to technology, R&D, intellectual property, and capital [60], the government's effect on MSMEs is proportional to their authority in the entrepreneurial environment.

In previous study, stakeholder engagement has often been examined as an integral part of business strategy, emphasizing how stakeholder involvement can impact company performance [[61], [62], [63]]. Some studies may have also discussed its positive effects on corporate reputation and relationships with customers, investors, and other stakeholders [[64], [65], [66]]. Meanwhile, the concept of costless signaling has become an intriguing topic in companies' efforts to communicate effectively without incurring significant costs. Although the previous literature may have explored some costless signaling methods used by organizations, a more in-depth analysis is needed into how companies manage costless signaling and its impact on stakeholders' perceptions and responses. Costless signaling refers to how companies communicate their strategic information without incurring significant costs. This can involve the use of social media, online promotions, or other effective methods without utilizing substantial resources.

In the other hand, study on digital adaptability may have highlighted how organizations adopt digital technology to enhance their performance. Digital adaptability pertains to a company's ability to adapt to digital technology and leverage it in their operations. This includes the use of digital tools, online platforms, and cutting-edge technology to enhance efficiency and competitiveness. However, the interconnection of digital adaptability with stakeholder engagement and costless signaling has not been fully explored. Research gaps in previous studies may lie in a deeper understanding of how these three concepts are interconnected and mutually influence each other. Therefore, this research aims to fill these gaps and provide new insights into the roles of stakeholder engagement, costless signaling, and digital adaptability in the context of corporate sustainability.

The novelty of this research lies in its focus on integrating three key factors, namely stakeholder engagement, costless signaling, and digital adaptability, within the context of sustainable performance in MSMEs. While previous studies have examined these factors separately, this research expands our understanding by combining them into a comprehensive research framework. Thus, it offers a new contribution to understanding the dynamics of stakeholder engagement, costless signaling, and digital adaptability that impact sustainable performance in MSMEs. This study aims to investigate the relationship between stakeholder engagement, costless signaling, digital adaptability, and sustainable performance in MSMEs. The study aims to understand better the factors influencing sustainable performance in MSMEs and examine whether digital adaptability moderates the relationship between costless signaling and sustainable performance.

## 2 Literature review

### 2.1 Micro, small, and medium enterprises

According to Suyanto & Kurniawan [67], Micro, small, and medium enterprises (MSMEs) is Micro Enterprises are productive businesses owned by individuals or sole proprietorships that meet Micro Enterprise criteria as regulated by the Law, namely having a net worth of up to Rp50,000,000.00 (fifty million Indonesian Rupiah) excluding land and building assets; or having annual sales of up to Rp300,000,000.00 (three hundred million Indonesian Rupiah). Small Enterprises are independent productive economic businesses conducted by individuals or entities that are not subsidiaries or branches of Medium or Large Enterprises, meeting Small Enterprise criteria as defined by the Law, with a net worth of more than Rp50,000,000.00 (fifty million Indonesian Rupiah) up to Rp500,000,000.00 (five hundred million Indonesian Rupiah) excluding land and building assets; or having annual sales of more than Rp300,000,000.00 (three hundred million Indonesian Rupiah) up to Rp2,500,000,000.00 (two billion five hundred million Indonesian Rupiah). Medium Enterprises are independent productive economic businesses conducted by individuals or entities that are not subsidiaries or branches of Small or Large Enterprises, with a net worth or annual sales as regulated by the Law, namely having a net worth of more than Rp500,000,000.00 (five hundred million Indonesian Rupiah) up to Rp10,000,000,000.00 (ten billion Indonesian Rupiah) excluding land and building assets; or having annual sales of more than Rp2,500,000,000.00 (two billion five hundred million Indonesian Rupiah) up to Rp50,000,000,000.00 (fifty billion Indonesian Rupiah).

Furthermore, according to the European Commission, a micro-enterprise is a business with fewer than 10 employees and annual turnover or balance sheet below €2 million. Small enterprises have fewer than 50 employees and annual turnover or balance sheet below €10 million. Meanwhile, medium-sized enterprises have fewer than 250 employees and annual turnover below €50 million or a balance sheet below €43 million. This definition is important as it determines eligibility for financial programs and support both at the EU and national levels. Since January 1, 2005, this definition has been in effect and has impacted many aspects of the MSME world [68].

The relationship between stakeholder engagement and costless signaling.

Spence [69] proposed the first signaling theory, which suggested that messages transmitted by businesses might be expensive (costly signaling) or inexpensive (costless signaling) [70]. Better for the firm as it will encourage potential investors to relay this knowledge so that the company's stock price continues to rise. In buyback announcements, costless signaling is a signal, but the firm does not repurchase, whereas expensive signaling is a signal, and the company does repurchase. Good firms may separate themselves from bad companies by costless signaling by drawing the attention and observation of investors or speculators. Still, bad companies will not want to take risks that investors will inspect [17]. The speculator carries costs incurred in costless signaling, but the firm bears costs incurred in costly signaling. As a result, undervalued firms choose costless signaling. Nevertheless, costless signaling occurs only in unusual conditions since the ability to attract speculators' interest is governed by whether it provides sufficient explanations for investment to research the business and the economic benefit may receive from private information concerning the company. Benefits will be considered if there is increased firm-specific risk concerning the business's worth, limited public information about the company in the market, or the company is significantly undervalued [71]. Stakeholder engagement involves actively involving stakeholders in decision-making processes and considering their interests, while costless signaling utilizes low-cost methods to share information with stakeholders [46]. By combining these two elements, organizations can build strong relationships with stakeholders and effectively communicate their sustainability initiatives without significant financial investments. This integrated approach promotes transparency, trust, and collaboration, ultimately contributing to improved sustainability performance and reputation for the organization. The hypothesis proposes as follows.

H1 stakeholder engagement relates on costless signaling

### 2.2 The relationship between stakeholder engagement and sustainable MSME performance

Salvioni & Almici [48] assessed stakeholder engagement plays a critical role in the performance of sustainability. By actively involving and collaborating with stakeholders such as employees, customers, suppliers, and local communities, these enterprises can benefit in various ways [6]. Through collaboration and input, stakeholder engagement allows for a comprehensive understanding of sustainability challenges and helps in developing effective strategies [27]. It also enhances the reputation and brand value of the enterprise, attracting conscious consumers and investors. Stakeholder engagement fosters innovation and continuous improvement, driving sustainability performance and identifying emerging risks and opportunities. Furthermore, engaged employees contribute to increased productivity and operational efficiency [72]. Overall, stakeholder engagement empowers sustainability-focused food and beverage small enterprises to achieve positive outcomes, address challenges, and drive long-term success in sustainability. The hypothesis proposes as follows.

H2 stakeholder engagement relates on sustainable MSME performance

The relationship between costless signaling and sustainable company performance and its mediates.

Costless signaling can help organizations or individuals improve their perception among stakeholders regarding their commitment to sustainable practices. Organizations can build a positive image as socially and environmentally responsible players by communicating sustainable actions and values through costless signaling. It can increase trust, attractiveness, and stakeholder loyalty toward the organization. It can invite greater stakeholder participation and engagement in sustainability efforts. For example, by leveraging social media as a costless signaling tool, organizations can encourage interactions and discussions with stakeholders, which in turn can strengthen relationships and gain valuable perspectives in sustainability endeavors. On the other hand, Effective costless signaling can enhance an organization's competitiveness in sustainability-conscious markets. Consumers and investors often choose products and services from organizations that clearly and credibly communicate their commitment to sustainability practices through costless signaling. Thus, costless signaling can provide a competitive advantage for organizations in gaining a larger market share. In addition, Costless signaling can help reinforce the identity and culture of an organization toward sustainability. Organizations can consistently communicate sustainability values and practices to their members by utilizing costless signaling, influencing sustainable norms, behaviors, and attitudes. It is important to note that real and consistent actions in sustainable practices should support costless signaling. Isolated costless signaling that does not reflect the organization's real efforts can lead to distrust and backlash from stakeholders. Therefore, effective costless signaling should be backed by concrete actions consistent with the message. Costless signaling can effectively influence stakeholder perceptions, gain competitive advantages, and reinforce organizational commitments and sustainable practices. However, the relationship between costless signaling and sustainable performance should be considered holistically and within the specific context of the involved organizations or individuals.

The mediating effect of costless signaling suggests that it acts as a mechanism through which stakeholder engagement translates into improved sustainable company performance. By effectively communicating sustainability initiatives and values through costless signaling, organizations can enhance their reputation, attract socially conscious customers and investors, and drive positive outcomes regarding environmental impact, social responsibility, and financial performance. Organizations must recognize the significance of costless signaling as a mediating factor and actively invest in strategies and tools that facilitate effective communication and stakeholder engagement. By doing so, they can leverage stakeholder engagement to improve sustainable company performance and create long-term value for the organization and its stakeholders. The hypothesis proposes as follows.

H3 Costless signaling relates on sustainable MSME performance

H4 Costless signaling mediate stakeholder engagement and sustainable MSME performance

#### 2.2.1 Magnitude of digital adaptability as moderator

High digital adaptability enhances stakeholder engagement by enabling real-time communication, personalized interactions, and co-creating sustainability initiatives, fostering relationships and commitment. It also facilitates costless signaling through various digital tools and platforms, reaching a wider audience and amplifying sustainability efforts. Conversely, low digital adaptability hinders stakeholder engagement and limits the effectiveness of costless signaling. Therefore, organizations should prioritize digital adaptability by investing in skills, infrastructure, and strategies aligned with stakeholder engagement and costless signaling. It enhances stakeholder relationships, strengthens sustainability messaging, and improves outcomes for the organization. Digital adaptability is crucial in moderating the relationship between stakeholder engagement, costless signaling, and sustainable company performance. The hypothesis proposes as follows.

H5 Magnitude of digital adaptability moderate stakeholder engagement and sustainable MSME performance

H6 Magnitude of digital adaptability moderate costless signaling sustainable MSME performance

**Fig. 1:**
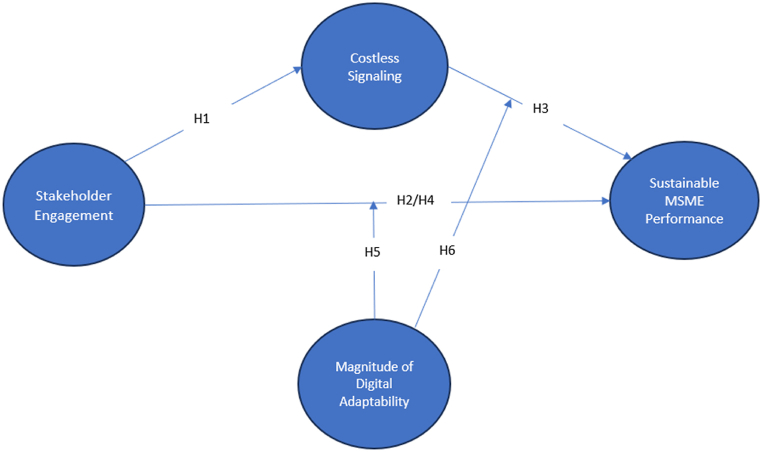
Conceptual framework.

Based on the literature review and hypothesis development, the conceptual framework of this study can be seen in Fig. 1. The framework depicts the relationships and variables that will be examined in this research. The main constructs included in this framework are costless signaling, digital adaptability, stakeholder engagement, and sustainable company performance. Stakeholder engagement is represented as the independent variable that is expected to have a positive influence on both costless signaling and sustainable company performance. Digital adaptability is proposed as a moderator that affects the relationship between stakeholder engagement, costless signaling, and sustainable company performance. Costless signaling is also considered a mediator mediating the relationship between stakeholder engagement and sustainable company performance. This framework provides a visual representation of the proposed relationships and will guide the analysis and interpretation of the research findings.

## 3 Methodology

This study adopts a quantitative research design to investigate the interplay between stakeholder engagement, costless signaling, and sustainable MSME performance, focusing on the moderating role of the magnitude of digital adaptability. The research design entails collecting data through questionnaires and statistical analysis to examine the proposed hypotheses. In terms of the population, this study targets food and beverage MSMEs operating in Banten Province. Banten has been strategically chosen as the focal point for research due to several compelling factors. Renowned for its rich cultural culinary diversity, the region showcases a spectrum of traditional and modern dishes, promising profound insights into the intricate landscape of Micro, Small, and Medium Enterprises (MSMEs) in the culinary and food industry. Economically significant, Banten plays a pivotal role with substantial numbers and impact of MSMEs operating in this sector, making it an ideal locale to gain valuable insights into the challenges and opportunities faced by these enterprises. Practical considerations also contribute to the decision, as the accessibility and availability of resources for researchers in the region can significantly impact the study. Furthermore, existing connections or partnerships in Banten streamline data collection and foster collaboration with local businesses. Although Banten may not encapsulate the entirety of Indonesia's culinary and food MSMEs, it serves as a poignant case study. The findings derived from Banten are anticipated to offer pertinent insights that can be generalized and provide a solid foundation for broader national-level research in this domain.

In an effort to delve deeper into the specific characteristics of Micro, Small, and Medium Enterprises (MSMEs) in the food and beverage sector in Banten, we present a more detailed analysis. The focused analysis includes an exploration of the significant economic contributions, an assessment of the evolving level of innovation, and a profound understanding of the various challenges faced by businesses at the local level. By providing this more detailed insight, it is expected that readers can sense the urgency and importance of selecting a sample from this sector within the context of our research.

The refinement of our research objectives involves a more detailed identification of growth patterns in MSMEs in the food and beverage sector. The breakdown includes a deeper understanding of the evolutionary dynamics of MSMEs in this sector. Additionally, we will elaborate more extensively on how this research contributes practically and in-depth, addressing specific challenges within the industry and shedding light on potential opportunities. With this more detailed breakdown, we hope our research contribution becomes more specific and relevant to the development of pertinent literature.

This section will be enriched to highlight more sharply the potential impact of the research on the general understanding of MSMEs and the food and beverage industry. More detailed explanations will depict the contribution of research findings to the formulation of more effective policies and sustainable industry practices. We will also elaborate more extensively on how the new insights gained from this research can provide direct benefits to stakeholders at the local and national levels. With this enriched potential impact, we anticipate our research will make a substantial and sustainable contribution to the development of MSMEs in the food and beverage sector in Banten.

The sample will be selected randomly using an appropriate sampling method, and the sample size will be determined based on statistical considerations to ensure adequate confidence levels and reliable outcomes amount 145 participants. In this study, the selection of units/analysis and our sampling framework is guided by our primary objective, which is to gain a profound understanding of the contributions of Micro, Small, and Medium Enterprises (MSMEs) in the food and beverage sector in Banten. The decision to focus on Banten as the research location is based on the unique characteristics of this region in terms of culinary diversity and its role as a significant economic hub. We carefully considered the selection criteria for MSMEs, encompassing various business sizes, operational durations, and types of enterprises to represent the diversity within the food and beverage industry, ranging from traditional eateries to innovative startups.

The choice of units/analysis is directed by the specific research goal of identifying growth patterns, innovations, and challenges faced by MSMEs in this sector. We aimed to select units/analysis that could provide an accurate representation of the diverse characteristics and experiences of MSMEs in the Banten region, intending to offer insightful findings relevant to our research questions.

Furthermore, our sampling framework is not solely based on the general characteristics of MSMEs but also considers the specific context of Banten. This choice is expected to make a substantial contribution to our understanding of the crucial role of MSMEs in the food and beverage industry in this region. Therefore, we believe that the selection of units/analysis and the sampling framework will support the achievement of our research goals and make a meaningful contribution to the relevant literature.

The research variables encompass the following components. Firstly, the independent variable is stakeholder engagement using five items Ferreira et al. [46] adopted. Secondly, the mediating variable is costless signaling using five things adopted by Colombo [70], and the moderating variable is the magnitude of digital adaptability users, five items adopted by Ferraro & Cristiano [21]. Lastly, the dependent variable is sustainable MSME performance using five things that Ortiz-de-Mandojana & Bansal [71] assumed. Data collection will use tailored questionnaires to capture stakeholder engagement levels, costless signaling methods, and sustainable MSME performance. The questionnaires will be administered to respondents who are either owners or representatives of MSMEs. The research procedure encompasses several stages. It begins with research preparation, which involves conducting a literature review, developing research instruments, and addressing ethical considerations. Following this, data collection will occur by distributing the questionnaires to the selected MSMEs within a specified timeframe. Confirming that questionnaire consent has been obtained from all participants for our study. Subsequently, the collected data will be analyzed using the statistical software Smart PLS to examine the relationships between variables and test the research hypotheses.

## 4 Findings

We assessed the validity of the indicators using the convergent technique, looking at the external loading factor values. A range of 0.50–0.70 for the loading factor is sufficient in early exploratory investigations. In this study, all indicators had extreme loading values greater than 0.70, indicating good convergent validity. We then evaluated the discriminant validity of each variable by comparing the square root of the average variance extracted (AVE) for each latent factor with the correlation coefficients between other elements in the model. It helped us determine if the variable could distinguish between different groups [73]. The value of the variable indicators is determined through the utilization of composite reliability in the very last phase. Results were judged reliable whenever the composite reliability and Cronbach's alpha were significantly higher than 0.7 The reliability of the variable indicators was determined using composite reliability. The indicators were considered reliable if both the composite reliability and Cronbach's alpha values were above 0.70 [74]. (see Table 1).

**Table 1:** Explanatory result.

Construct	Items	Outer Loading	Cronbach's Alpha	rho_A	CR	AVE
Costless Signaling	COST1	0.833	0.829	0.831	0.880	0.796
	COST2	0.796				
	COST3	0.739				
	COST4	0.747				
	COST5	0.739				
Magnitude of Digital Adaptation	MDA1	0.702	0.920	0.987	0.935	0.743
	MDA2	0.867				
	MDA3	0.907				
	MDA4	0.912				
	MDA5	0.905				
Stakeholder Engagement	SHE1	0.937	0.966	0.97	0.974	0.881
	SHE2	0.964				
	SHE3	0.928				
	SHE4	0.967				
	SHE5	0.894				
Sustainable MSME performance	SSP1	0.873	0.908	0.911	0.932	0.734
	SSP2	0.872				
	SSP3	0.903				
	SSP4	0.884				
	SSP5	0.740				

The composite reliability calculation showed values ranging from 0.831 to 0.987 (above 0.70), indicating that the indicators of the variable were reliable. Similarly, Cronbach's alpha scores ranged from 0.734 to 0.881, exceeding the threshold of 0.70, further confirming the reliability of the indicators and their freedom from errors [74].

Analyzing the P-Values provides insights into the level of significance associated with accepting a hypothesis. The validation of the study's hypothesis relies on P-Values being less than 0.05. In SmartPLS, a bootstrapping procedure is conducted on a reliable and valid model that meets the requirements to obtain the P-value of the model. The outcomes of the bootstrapping process are presented in Table 2.

**Table 2:** Path coefficient result.

Hypothesis	Construct^a^	Original Sample	Standard Deviation	T Statistics	P Values	Result
H1	SHE - > COST	0.468	0.125	3.753	0.000	Accepted
H2	SHE - > SSE	0.208	0.081	2.562	0.011	Accepted
H3	COST - > SSP	0.560	0.063	8.856	0.000	Accepted

a SHE=Stakeholder Engagement; COST=Costless Signaling; SSP = sustainable MSME performance.

**Fig. 2:**
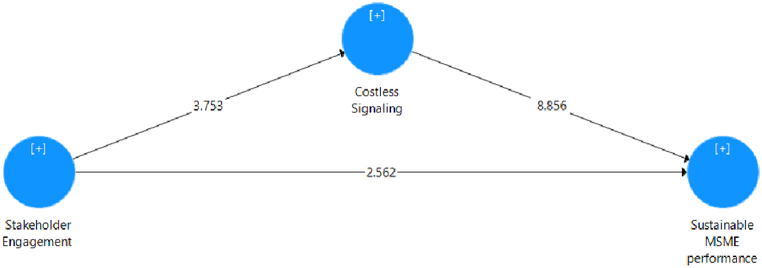
Path analysis.

Based on the path coefficient in Fig. 2 and Table 2 obtained, which is 0.468, and a P-Value of 0.000 < 0.05, it can be concluded that stakeholder engagement significantly impacts costless signaling, thus providing support for the acceptance of H1. Our descriptive test highlights that the level of stakeholder engagement in this context is well-measured, with a high average value. This indicates that in our research sample, companies tend to actively involve stakeholders in decision-making and policy formulation. When we compare this finding with the statistical results showing a significant impact, it can be inferred that not only is stakeholder engagement generally high, but it also has significant consequences for costless signaling. In other words, these results depict that stakeholder engagement is not merely a common practice undertaken by companies; it also has strong positive implications for a company's ability to engage in costless signaling. Therefore, these findings provide a more comprehensive and holistic foundation for the significant contribution of stakeholder engagement in the context of costless signaling, going beyond mere statistical results.

10.13039/100014337Furthermore, the correlation between stakeholder engagement and sustainable MSME performance is 0.208 with a P-Value of 0.011 < 0.05, indicating a significant relationship between the two variables and supporting H2. Through descriptive analysis, we observed that the levels of stakeholder engagement and sustainable MSME performance in this context show relatively high values, reflecting that companies tend to actively involve stakeholders in operational activities and strategic decision-making. When examining the statistical results showing a significant correlation, we can interpret that not only is stakeholder engagement generally high, but it also correlates positively with improved sustainable MSME performance. In other words, these findings not only state that there is a significant relationship between stakeholder engagement and sustainable MSME performance but also provide a deeper understanding of how this engagement can positively contribute to achieving sustainable performance. Therefore, these results form a richer and more comprehensive foundation to support hypothesis H2, involving broader aspects than just statistical figures alone.

Similarly, the correlation between costless signaling and sustainable MSME performance is 0.560, with a P-Value of 0.000 < 0.05, indicating a significant relationship. Accepting H3 suggests that costless signaling positively influences sustainable MSME performance. Through descriptive analysis, we noted that the degree of costless signaling and sustainable MSME performance in this particular context exhibits robust values. This observation implies that companies in the study tend to effectively communicate strategic information without incurring significant costs, aligning with practices conducive to sustainable performance. When we examine the statistical results, indicating a substantial correlation, we can infer that not only is costless signaling prevalent, but it also has a notable positive impact on enhancing sustainable MSME performance. In essence, these findings not only affirm the existence of a significant relationship between costless signaling and sustainable MSME performance but also provide a richer understanding of how effective signaling practices can positively shape and contribute to sustained performance. Therefore, accepting H3 goes beyond acknowledging statistical significance, offering insights into the practical implications and broader dynamics of costless signaling on sustainable MSME performance. To investigate indirect effects, such as mediation and moderation, please refer to Table 3.

**Table 3:** Indirect test result.

Hypothesis	Construct^a^)	Original Sample	Standard Deviation	T Statistics	P Values	Result
H4	SHE - > COST - > SSP	0.262	0.081	3.230	0.001	Accepted
H5	MDA^a^SHE - > SSP	−0.136	0.065	2.098	0.036	Accepted
H6	MDA^a^COST- > SSP	0.004	0.057	0.078	0.938	Rejected

a SHE=Stakeholder Engagement; COST=Costless Signaling; MDA = Magnitude of Digital Adaptation; SSP = sustainable MSME performance.

The results indicate that costless signaling positively impacts both stakeholder engagement and sustainable MSME performance (t = 3.230 > 1.96). It suggests that stakeholder engagement mediates between costless signaling and sustainable MSME performance, supporting the acceptance of H4. Upon delving into the descriptive analysis, we observed that the positive impact of costless signaling resonates not only with statistical significance but is also evident in the effective involvement of stakeholders and enhanced sustainable MSME performance within our research sample. The robust t-value further emphasizes the substantial influence of costless signaling on both variables. In essence, these findings imply that companies employing costless signaling practices not only foster stakeholder engagement but also contribute to improved sustainable MSME performance. This aligns with the mediation hypothesis (H4), indicating that stakeholder engagement plays a pivotal role in translating the positive effects of costless signaling into sustained performance. Therefore, the acceptance of H4 expands our understanding beyond statistical significance, shedding light on the intricate relationship dynamics and the practical implications of costless signaling on stakeholder engagement and sustainable MSME performance.

MSMEs with a high magnitude of digital adaptability will have high stakeholder management relationships with sustainable MSMSE performance and vice versa. As can be seen in the equation line drawing, the magnitude of digital adaptability at-1 increases; instead, it increases the magnitude of digital adaptability at-1. The magnitude of digital adaptability weakens the relationship between stakeholder management and sustainable MSMSE performance. Even though the effect is very small, as seen in the equation line drawing, which tends to be straight or there is no change, it can be concluded that the magnitude of digital adaptability does not strengthen or weaken the relationship between costless signaling and sustainable MSMSE performance.

In the moderation test, with a T Statistics value of 2.098 > 1.96 and a significance level of 5 %, the magnitude of digital adaptability significantly influences stakeholder engagement and sustainable MSME performance. Therefore, the magnitude of digital adaptability moderates the relationship between stakeholder engagement and sustainable MSME performance; thus, Hypothesis 5 is accepted. However, the original sample value shows a negative direction, indicating that the magnitude of digital adaptability weakens the relationship between stakeholder engagement and sustainable MSME performance. Delving into the descriptive layer, we uncover a notable aspect. While the statistical outcome indicates a significant moderation effect, the original sample value shows a negative direction. This intriguing observation suggests that, in the context of our research sample, the magnitude of digital adaptability introduces a nuanced dynamic. It not only significantly influences the relationship between stakeholder engagement and sustainable MSME performance but does so by weakening this relationship. In essence, these findings go beyond the statistical inference, offering a richer understanding. They imply that the impact of digital adaptability is not uniformly positive; rather, it introduces a moderating influence that alters the nature of the relationship between stakeholders and sustainable MSME performance. Accepting Hypothesis 5 acknowledges this nuanced perspective, shedding light on the intricate interplay of factors and the practical implications of digital adaptability in shaping the dynamics between stakeholders and MSME sustainability.

Additionally, the magnitude of digital adaptability and the relationship between costless signaling and sustainable MSME performance is very weak, with a T Statistics value of 0.078 < 1.96 and a significance level of 5 %. Therefore, the implications suggest that the magnitude of digital adaptability does not strengthen or weaken the relationship between costless signaling and sustainable MSME performance, leading to the rejection of Hypothesis 6. Through descriptive analysis, we found that the magnitude of digital adaptability and the relationship between costless signaling and sustainable MSME performance tend to exhibit a weak connection. This reflects that, in our research context, the factor of digital adaptability does not significantly contribute to strengthening or weakening the impact of costless signaling on sustainable MSME performance. Thus, these results go beyond statistical values and highlight the practical weakness of the correlation between these variables. The rejection of Hypothesis 6 adds a deeper understanding that in the context of digital adaptability, there is no significant change in the dynamics of the relationship between costless signaling and sustainable MSME performance.

The rejection of Hypothesis 6, indicating a significant interaction effect between the magnitude of digital adaptability and the relationship between costless signaling and sustainable MSME performance, is based on a combination of statistical analysis and contextual reasoning. Statistically, the T Statistics value of 0.078 falling below the critical threshold of 1.96, coupled with a significance level of 5 %, indicates a lack of robustness in the relationship. This implies that the observed magnitude of digital adaptability does not exert a substantial influence on either strengthening or weakening the connection between costless signaling and sustainable MSME performance. Contextually, the descriptive analysis further supports this rejection by revealing a weak correlation between the variables. In the context of the study, digital adaptability does not exhibit a pronounced impact on altering the dynamics of how costless signaling influences sustainable MSME performance. The limited practical significance observed in the descriptive findings aligns with the statistical outcome, contributing to the rejection of Hypothesis 6. Therefore, the rejection of Hypothesis 6 is not solely based on statistical values but is substantiated by the practical implications derived from both statistical analysis and a nuanced understanding of the observed weak correlations in the context of digital adaptability's impact on the relationship between costless signaling and sustainable MSME performance.

## 5 Discussions

Stakeholder engagement positively impacts costless signaling in sustainability initiatives (H1 accepted). Engaged stakeholders are likelier to promote and communicate the organization's sustainability efforts through various communication channels without significant financial investments. They act as advocates, spreading positive messages and information about the organization's sustainable practices to a wider audience. Stakeholder engagement creates a supportive network that amplifies costless signaling, increasing awareness and generating interest in sustainability. Moreover, engaged stakeholders feel a sense of ownership and commitment, voluntarily sharing their experiences and success stories related to sustainability, enhancing the organization's reputation. Cultivating strong relationships with stakeholders and involving them in sustainability initiatives allows organizations to leverage costless signaling effectively, effectively communicating their commitment to sustainability and engaging a broader audience.

Stakeholder engagement significantly impacts the sustainable performance of MSMEs (H2 accepted). When stakeholders are actively involved in the sustainable activities of MSMEs, they can make positive contributions that influence the business's overall performance. Engaged stakeholders may provide MSMEs support, resources, and valuable insights, leading to improved sustainability practices, operational efficiency, and comprehensive business performance. Their involvement can also enhance the reputation and credibility of the MSMEs, attracting socially conscious customers, investors, and potential business partners. Furthermore, engaged stakeholders may advocate for the MSMEs, spreading positive word-of-mouth and increasing awareness and market visibility. Therefore, stakeholder engagement plays a crucial role in driving the sustainable performance of MSMEs by fostering collaboration, support, and shared responsibility toward sustainable goals.

Hypothesis 3 (H3) ‘s acceptance indicates that costless signaling enables MSMEs to enhance their reputation, attract environmentally conscious customers, and build stakeholder trust. It also contributes to increased market visibility, customer loyalty, and competitiveness in the sustainability realm. MSMEs can drive their sustainability efforts by reaching a broader audience and engaging stakeholders without significant financial investments. In summary, this hypothesis emphasizes the importance of costless signaling in promoting sustainable performance for MSMEs. MSMEs can communicate their sustainable practices, generate stakeholder interest and support, and achieve favorable sustainability outcomes.

H4 accepted, it implies that costless signaling as mediating between stakeholder engagement and the sustainable performance of MSMEs. When stakeholders are actively engaged and involved in an MSME's sustainability initiatives, their support and advocacy can be effectively communicated through costless signaling methods. Costless signaling acts as a bridge, transmitting the positive impact of stakeholder engagement on sustainable performance. MSMEs can amplify their sustainability messages, reach a wider audience, and enhance their sustainable performance outcomes by utilizing various costless signaling channels such as social media, word-of-mouth, and online platforms. The mediating effect of costless signaling implies that stakeholder engagement alone may not directly influence sustainable performance. Still, effective communication and dissemination of stakeholder engagement efforts through costless signaling contribute to improved sustainable performance for MSMEs.

The magnitude of digital adaptability weakens the relationship between stakeholder engagement and sustainable MSME performance implies that when the level of digital adaptability is high, it can reduce the impact of stakeholder engagement on sustainable performance in MSMEs. In other words, even with actively engaged stakeholders, MSMEs may only achieve significant improvements in sustainability outcomes if they have the digital capabilities and infrastructure needed to leverage stakeholder engagement effectively. A high magnitude of digital adaptability indicates that MSMEs have adopted digital technologies, tools, and platforms to enhance their sustainability practices and interact with stakeholders. However, if digital adaptability is not effectively aligned with stakeholder engagement efforts, it can weaken the relationship between stakeholder engagement and sustainable performance in MSMEs. This weakening effect is because MSMEs with high digital adaptability may become overly reliant on digital communication channels and must pay more attention to building strong relationships and direct interactions with stakeholders. While digital tools enable efficient and widespread communication, they may need more personal touch and depth of engagement to build stakeholder trust, collaboration, and commitment. Furthermore, if MSMEs prioritize quantity over quality of stakeholder engagement through digital media, the meaningfulness and impact of attention can be affected. Stakeholders may perceive such engagement efforts as superficial or insincere, leading to decreased support, advocacy, and, ultimately, weaker connections between stakeholder engagement and sustainable performance. To address this, MSMEs need to maintain a balance between digital adaptability and meaningful stakeholder engagement. They should strategically use digital tools and platforms to complement and enhance direct interactions with stakeholders rather than replace them. Building strong relationships, understanding stakeholder expectations, and involving them in decision-making processes remain essential for driving sustainable performance in MSMEs. In conclusion, while digital adaptability can provide opportunities for MSMEs to interact with stakeholders, it must be integrated and aligned with meaningful stakeholder engagement efforts to maximize its positive impact on sustainable performance. MSMEs need to consider the quality, depth, and authenticity of stakeholder engagement, in line with their digital capabilities, to achieve meaningful and sustainable.

The magnitude of digital adaptability does not moderate the relationship between costless signaling and sustainable MSME performance. The level of digital adaptability does not significantly influence the impact of costless signaling on sustainable performance in MSMEs. In other words, regardless of the degree of digital adaptability, the relationship between costless signaling and sustainable MSME performance remains consistent. It implies that the effectiveness of costless signaling methods in driving sustainable performance is relatively unaffected by the level of digital adaptability in MSMEs. Whether an MSME has high or low digital adaptability, communicating sustainability messages and engaging stakeholders through costless signaling methods can still positively impact sustainable performance outcomes. The absence of a moderating effect of digital adaptability suggests that the utilization of costless signaling methods, such as social media, word-of-mouth, and online platforms, remains effective in enhancing sustainable MSME performance regardless of the digital capabilities and infrastructure available to the organization. It emphasizes the importance of leveraging costless signaling strategies to effectively communicate sustainability efforts and engage stakeholders, irrespective of the digital landscape in which the MSME operates. In summary, the level of digital adaptability does not influence the relationship between costless signaling and sustainable MSME performance. Regardless of the digital capabilities of an MSME, the utilization of costless signaling methods can still positively impact sustainable performance outcomes.

## 6 Conclusion

The study findings confirm the positive impact of stakeholder engagement on costless signaling in sustainability initiatives, with engaged stakeholders acting as advocates and amplifying the organization's sustainability efforts through various communication channels. It fosters increased awareness and interest in sustainability, contributing to enhanced reputation and commitment. Moreover, stakeholder engagement significantly influences the sustainable performance of MSMEs by fostering collaboration and support, leading to improved practices and overall business performance. Costless signaling plays a crucial role in enhancing reputation, attracting environmentally conscious customers, and increasing market visibility and competitiveness in sustainability. It acts as a mediator between stakeholder engagement and sustainable performance, effectively transmitting the positive effects of engagement. However, the study highlights that the magnitude of digital adaptability weakens the relationship between stakeholder engagement and sustainable MSME performance, emphasizing the need for alignment between digital capabilities and meaningful stakeholder engagement to maximize sustainable outcomes. Conversely, digital adaptability does not moderate the relationship between costless signaling and sustainable MSME performance, indicating the consistent effectiveness of costless signaling methods regardless of digital infrastructure. These findings underscore the importance of integrating digital tools with meaningful stakeholder engagement to drive sustainable performance in MSMEs effectively.

## 7 Implications

The findings of this research carry significant implications for MSMEs business practices and sustainability efforts. The results suggest that active stakeholder engagement plays a crucial role in increasing sustainability awareness and creating a supportive network that strengthens costless signaling. Additionally, stakeholder engagement positively influences the sustainable performance of MSMEs by contributing positively, offering support, and enhancing the company's reputation. The study also highlights the mediating role of costless signaling in effectively communicating the positive impact of stakeholder engagement on sustainable performance. However, the observed high level of digital adaptability appears to weaken the relationship between stakeholder engagement and sustainable performance. Therefore, MSMEs need to strike a balance between digital adaptability and direct stakeholder engagement to maximize its positive impact. These implications provide a strategic foundation for MSMEs to enhance their sustainability through stakeholder engagement and the utilization of costless signaling, considering digital adaptability as a critical factor in this dynamic.

### 7.1 Limitations and future research

This research has several limitations that need to be considered. First, this study only focuses on the relationship between stakeholder involvement, costless signaling, and sustainable performance in MSMEs. Other factors may also influence sustainable performance, such as the organization's economic, regulatory, and internal aspects, which can be the focus of further research. Second, this study uses a quantitative approach using survey data. Future research can complement these findings using a qualitative system or mixed methods to understand better stakeholder experiences and perceptions regarding engagement and costless signaling in MSMEs. Third, the research is conducted in a specific context, such as a geographical area or industry. Future research can broaden geographic and industry coverage to obtain broader generalizations about the relationship between stakeholder engagement, costless signaling, and sustainable performance in MSMEs. With these limitations in the study, future research can more deeply examine the factors influencing sustainable performance in MSMEs and develop more effective strategies to drive sustainability in the sector.

## Data availability statement

The data associated with this study has not been deposited into a publicly available repository. As the research did not utilize any specific dataset or raw data, no data sharing is applicable. All necessary information and data supporting the findings of this study are included within the manuscript and its supplementary materials. Any inquiries regarding the data used in this study can be directed to the corresponding author for further clarification.

## CRediT authorship contribution statement

**Uli Wildan Nuryanto:** Writing – original draft, Funding acquisition, Formal analysis, Data curation, Conceptualization. **Basrowi:** Writing – review & editing, Writing – original draft, Supervision, Methodology, Investigation, Writing – review & editing, Writing – original draft, Supervision, Methodology, Investigation. **Icin Quraysin:** Visualization, Resources, Project administration. **Ika Pratiwi:** Software, Project administration.

## Declaration of competing interest

The authors declare that they have no known competing financial interests or personal relationships that could have appeared to influence the work reported in this paper.
